# 

**Published:** 1882-05

**Authors:** 


					﻿Vol. II.
MAY, 1882=
No. 5.
THE
HOMEOPATHIC pH^lClAJJ,
A MONTHLY JOURNAL OF MEDICAL SCIENCE.
■“ Magna est veritas,. et prcevalebit.''
ZED. O’. LZEim. 3VE. X).
EDITOR.
CONTENTS:
(See inside of Cover.)
PHILADELPHIA:
No. 2109 CHESTNUT STREET.
LOKDO JST-
ALFRED HEATH & CO., Sole Agents, fob Great Britain,
114 Ebury Street, Eaton Square.
Yearly Subscription, $2.00, invariably in advance.
Entered at the Philadelphia Post-Office, as Second Class Mail Matter.
				

